# P03 - Mould allergy in children with allergic rhinitis in a seaside agricultural region

**DOI:** 10.1186/2045-7022-4-S1-P58

**Published:** 2014-02-28

**Authors:** Gavriela Feketea, Theodora Danidi

**Affiliations:** 1 General Hospital of Amaliada, Amaliada, Greece

## Objective

Allergic rhinitis (AR) is a major chronic respiratory disease due to its prevalence, impact on quality of life (QL), economic burden, link with asthma. During the last years, many countries have experienced an increase in the prevalence of respiratory allergy. A significant contribution in development of AR, in addition to house dust mite, cockroaches, dogs, cats, pollen and trees, seems to have mould allergens. *Alternaria* is predominantly an outdoor allergen favouring damp spots, and most indoor concentrations may derive from outdoor primary sources.

## Methods

We conducted a retrospective case-note review of all children seen in the allergy clinic during a nine months period, with a potential diagnosis of allergic rhinitis.

## Results

138 children, aged 3 to 16 years have been seen in our allergy outpatient department during a period of 9 month, for moderate / severe allergic rhinitis, according to ARIA classification. 53 from them were female and 85 male. Skin prick test (SPT) has been performed and they were tested for most common inhalant allergens for our region including wall pellitory, dust mite, grass mix, cat and dog, olive, *Aspergilus* species, *Cladosporium* and *Alternaria* standardized extract.

Twenty four children (17,4%) had a positive SPT to at least one of the three mould allergens investigated. Twelve children (8,7%) have been monosensitized only to *Alternaria* while 11 polysensitized ( 8%). Only one child was sensitized to *Cladosporium*, being sensitized to olive too. Previous studies showed for Greece, around 10% sensitization to moulds.

## Conclusion

The high frequency of sensitization to *Alternaria* (16,6%) in our region could be explained by the high level of humidity (range up to 95%) due to its location, near sea-coast. Another reason could be the intensive cultivation of tomato and other legume in our region while the indoor source remains an important one.

